# Distinct Proteasome Subpopulations in the Alveolar Space of Patients with the Acute Respiratory Distress Syndrome

**DOI:** 10.1155/2012/204250

**Published:** 2012-01-29

**Authors:** S. U. Sixt, R. Alami, J. Hakenbeck, M. Adamzik, A. Kloß, U. Costabel, P. R. Jungblut, B. Dahlmann, J. Peters

**Affiliations:** ^1^Klinik für Anästhesiologie und Intensivmedizin, Universität Duisburg-Essen, Universitätsklinikum Essen, 45122 Essen, Germany; ^2^Institut für Biochemie/CCM, Charité-Universitätsmedizin-Berlin, 13347 Berlin, Germany; ^3^Klinik für Pneumologie und Allergologie, Ruhrlandklinik, Universität Duisburg-Essen, 45239 Essen, Germany; ^4^Max Planck Institute for Infection Biology, Core Facility Protein Analysis, 13125 Berlin, Germany

## Abstract

There is increasing evidence that proteasomes have a biological role in the extracellular alveolar space, but inflammation could change their composition. We tested whether immunoproteasome protein-containing subpopulations are present in the alveolar space of patients with lung inflammation evoking the acute respiratory distress syndrome (ARDS). Bronchoalveolar lavage (BAL) supernatants and cell pellet lysate from ARDS patients (*n* = 28) and healthy subjects (*n* = 10) were analyzed for the presence of immunoproteasome proteins (LMP2 and LMP7) and proteasome subtypes by western blot, chromatographic purification, and 2D-dimensional gelelectrophoresis. In all ARDS patients but not in healthy subjects LMP7 and LMP2 were observed in BAL supernatants. Proteasomes purified from pooled ARDS BAL supernatant showed an altered enzyme activity ratio. Chromatography revealed a distinct pattern with 7 proteasome subtype peaks in BAL supernatant of ARDS patients that differed from healthy subjects. Total proteasome concentration in BAL supernatant was increased in ARDS (971 ng/mL ± 1116 versus 59 ± 25; *P* < 0.001), and all fluorogenic substrates were hydrolyzed, albeit to a lesser extent, with inhibition by epoxomicin (*P* = 0.0001). Thus, we identified for the first time immunoproteasome proteins and a distinct proteasomal subtype pattern in the alveolar space of ARDS patients, presumably in response to inflammation.

## 1. Introduction

The proteasome is a multicatalytic enzyme complex responsible for the degradation of the vast majority of intracellular proteins [1]. Proteasomes are involved in many basic cellular processes including the cell cycle, apoptosis, the stress response, and also in the regulation of immune and inflammatory responses [2–5]. The 26S proteasome consists of a catalytic 20S proteasome core and two 19S (cap) regulatory complexes.

The 20S proteasome itself is a 660–700 kDa [2, 6] multicatalytic proteinase complex with a cylinder-shaped structure arranged as four axially stacked heptametrical rings composed of seven *α* subunits (outer rings) and seven *β* subunits (inner rings), respectively [7]. The *α* type subunits have highly conserved N-terminal extensions which were proposed to have regulatory and targeting function [38]. The proteolytic activities of the 20S proteasome are described as trypsin, chymotrypsin, and peptidyl-glutamyl peptide hydrolyzing activity and are exclusively associated with the proteasome subunits *β*_1_, *β*_2_, and *β*_5_ [8, 9]. Five of the seven *β* type subunits are synthesized as precursor proteins with N-terminal propeptides that are cleaved off during 20S proteasome biogenesis [13–15] that is mediated by accessory proteins like the proteasome maturation protein (POMP) [10].

In cells exposed to IFN-*γ* or TNF-*α*, however, the standard *β* subunits can be replaced by so-called immuno-subunits *β*_1i_ (LMP2), *β*_2i_ (MECL-1), and *β*_5i_ (LMP7) that are incorporated cooperatively into newly synthesized proteasomes named “immunoproteasome”. In case that only partial replacement takes place “intermediate-type proteasomes” are formed [11].

The immunoproteasome is more likely to generate peptides with hydrophobic and basic C-terminal residues and less likely to generate peptides with acidic C-terminal residues [12–14]. These short peptides (8–10 amino acids) are subsequently translocated by the transporter associated with antigen processing (TAP) to the endoplasmic reticulum (ER), where a small part of them are loaded on major histocompatibility complex class-I molecules (MHC-I) and presented to cytotoxic T lymphocyte [15] on the cell membrane. Concomitant with immunoproteasome synthesis induced by IFN-*γ*, other components of the antigen presentation machinery, like TAP [16] or the proteasome activator 28 (PA28), are also upregulated, and a decreased concentration of standard intracellular 26S proteasome is observed [17].

While a prior paradigm was that the proteasome is located only intracellularly, it is now accepted that proteasomes can also be present extracellularly [10]. Recently, we have reported the presence of biologically active 20S proteasome in the extracellular alveolar space in healthy subjects [18] and in patients with the acute respiratory distress syndrome (ARDS) [19]. Since ARDS goes along with pulmonary inflammation [20], proinflammatory mediators [21, 22] like IFN-*γ* and TNF-*α* are produced, and the alveolar proteasomal system could be altered. Accordingly, we investigated whether alveolar proteasomal populations are changed in lung inflammation and whether immunoproteasomes are present in the alveolar space of ARDS patients.

## 2. Material and Methods

### 2.1. Patients and Clinical Procedures

Twenty-eight adult patients with severe ARDS (13 men, 15 women, mean age: 41 years ± 16 SD) were studied prospectively after approval of the Ethics Committee of the University of Essen Medical School. Characteristics of ARDS patients are depicted in Table 1. To assess disease severity, lung injury score [23], simplified acute physiology score (SAPS) [24], and sepsis-related organ failure assessment (SOFA) [25] were measured. Twenty-two patients (79%) had an ARDS of pulmonary origin, 50% underwent therapy with extracorporeal membrane oxygenation (ECMO), and overall in-hospital mortality was 53.6%.

Patients were considered to suffer from ARDS and eligible for BAL and blood sampling if they met the criteria proposed by Bernard [20]: PaO_2_/fraction of inspired oxygen (F_I_O_2_) ratio of ≤200 mmHg while on a positive end-expiratory pressure (PEEP) ≥10 cm H_2_O, bilateral radiographic pulmonary infiltrates, and no clinical evidence of left atrial hypertension or a pulmonary artery occlusion pressure of 18 mmHg or less. The bronchoalveolar lavage (BAL) was performed during sedation/anesthesia in the lung segment showing radiological consolidation and infiltration.

Ten adult subjects without lung disease (7 men, 3 women, mean age: 30 years ± 5) served as controls. They were free of lung, cardiac, infectious, and allergic disease, had no history of chemotherapy or radiation therapy, and they were nonsmokers. In these individuals, BAL and blood sampling were performed during local anesthesia.

### 2.2. Bronchoalveolar Lavage (BAL)

Within 24 h of admission, ARDS patients underwent BAL [26, 27] for routine workup of bacterial and viral infections. Four aliquots of warm (37°C) sterile isotonic saline (40 mL) were instilled via a bronchoscope wedged into a segmental bronchus and gently withdrawn. The BAL of healthy controls BAL was performed by instilling saline into the right middle or left lingular lob. A volume of greater than 50% was recovered, filtered through cotton gauze [28], and centrifuged (500 g, 10 min, 5°C). The BAL supernatant was immediately frozen using liquid nitrogen, stored at −80°C, and served as a sample of the extracellular alveolar fluid.

In the pellet, cell counts were assessed by counting an aliquot in a Neubauer chamber [28]. For cell differentiation, smears were air-dried and stained according to May-Grünwald-Giemsa [27]. The remaining cell pellet was immediately frozen in liquid nitrogen and stored at −80°C. After cell lysis, the cell pellet was ultracentrifugated (30000 g, 30 min, Beckman, München), and the upper portion of this centrifugation step was used for further analysis.

### 2.3. Blood Samples

To detect immunoproteasome proteins, if present, EDTA blood samples were drawn from all ARDS patients and healthy controls. Blood was centrifugated (500 g, 10 min, 5°C) to separate the supernatant (plasma) from cell pellet.

### 2.4. Measurements

#### 2.4.1. SDS-PAGE Gelelectrophoresis

SDS-PAGE was performed with Mini-Protean 3 Electrophoresis (Bio-Rad) with 15% gels according to [18]. 50 *μ*g protein per lane were applied. The molecular weight standard was SeeBlue Pre-Stained Standard obtained from Invitrogen.

#### 2.4.2. Detection of Immunoproteasome Proteins by Western Blots

To detect the presence of proteasomal proteins samples (50 *μ*g per lane) from 28 ARDS patients and from 10 healthy subjects the samples were subjected to SDS/PAGE and transferred to PVDF (BioRad) under semidry conditions with the use of a Trans-Blot Semi-Dry Electrophoretic Transfer Cell (BioRad). After blocking the PVDF membranes by incubation with TBS-Tween buffer (5% Tween 20, 150 mM NaCl, 20 mM Tris/HCl, pH 7.6) and StartingBlock Blocking Buffer (Pierce, Rockford) for 24 hours at 4°C, the membranes were incubated with rabbit polyclonal antibody to 20S proteasome subunit *β*_1i_ (LMP2) (Biomol International LP; PW 8840) (dilution 1 : 1000, 2 h, room temperature), rabbit polyclonal antibody to 20S proteasome subunit *β*_5i_ (LMP7) (dilution 1 : 2500, 2 h, room temperature), and with *rabbit* polyclonal antibody to proteasome activator 28 (PA28) (dilution 1 : 1000, 2 h, room temperature), as described elsewhere [29].

After washing with TBS-Tween buffer (5% Tween 20, 150 mM NaCl, 20 mM Tris/HCl, pH 7.6), the membranes were incubated (1 : 10000, 1 h, room temperature) with peroxidase-conjugated affinity-isolated goat anti-rabbit IgG (Sigma Aldrich). After washing, the chemoluminescence method was employed to detect the peroxidase activity using an ECL kit (SuperSignal West Pico Chemiluminescence Substrate, Pierce).

#### 2.4.3. Determination of Total Proteasome Concentration in BAL Supernatant

Proteasome concentration was measured [30] by ELISA in BAL supernatants of all ARDS and of all healthy subjects. Microtitration plates were coated overnight with mouse monoclonal antibody to 20S proteasome subunit *α*6 (HC2) (Biomol International L.P., Exeter, UK) 1 : 4500 in PBS (Invitrogen GmbH, Karlsruhe, FRG), pH 7.4. The BAL supernatants were diluted with an equal volume PBST-BSA (PBS, Tween 20, 0.1%, and 1% bovine serum albumin) and applied to each well for 3 hours at room temperature. All measurements were covered by the linear portion of the respective ELISA standard curve.

Standard curves were established for every microtitration plate using 20S proteasome protein standards (Biomol International L.P., Exeter, UK) of concentration ranging from 19.5 ng mL^−1^ to 2500 ng mL^−1^ (8 linear dilution steps). The 20S proteasome was diluted in PBS-T (PBS and Tween 20, 0.1%). The plates were washed once, and a rabbit polyclonal antibody (Biomol International L.P., Exeter, UK) to 20S proteasome (dilution 1 : 4000) was added for 2 hours at room temperature. Following another four washing steps peroxidase-conjugated mouse anti-rabbit IgG (Sigma-Aldrich, Saint Louis, USA) was used for antigen detection (incubation period: 1 h at room temperature). The bound antibodies were detected using tetramethylbenzidine (Sigma-Aldrich, Saint Louis, USA) as substrate. The reaction was stopped with sulphuric acid, and OD-values were determined at 450 nm. To exclude nonspecific binding, wells were filled with bovine serum albumin (Sigma-Aldrich, Saint Louis, USA), PBS, or PBS-T instead of BAL supernatant and incubated with the antibody. No reaction was observed under these control conditions.

#### 2.4.4. Purification of Proteasomes from BAL Supernatant

20S proteasomes from 5 patients with ARDS and from 5 healthy subjects were purified as described elsewhere [31]. All purification steps were performed at 4°C. To the pooled BAL supernatant from 5 ARDS patients the same volume of TEAD buffer (20 mM Tris/HCl, 1 mM EDTA, 1 mM NaN_3_, 1 mM DTT, pH 7.5) was added, and the mixture was homogenized by use of a Dounce homogenizer (20 strokes) under ice cooling. Undissolved material was separated by centrifugation (50 min at 20000 g). The supernatant was then subjected to a column (1 × 8 cm) of DEAE-Toyopearl 650S (TOSOH Biosep GmbH, Stuttgart, Germany) equilibrated with TEAD buffer. After washing the column with 50 mM NaCl/TEAD buffer, proteins bound to the resin were eluted with a linear gradient of 50–500 mM NaCl dissolved in TEAD buffer. Fractions of 1 mL were collected and tested for their proteasome activity with the fluorogenic substrate Suc-LLVY-AMC. Proteasome-containing fractions were then pooled, and 20S proteasomes were purified by successive chromatographies on Superose 6 (Pharmacia HR 10 × 30), Mono Q (HR 5/5) and Phenyl-Superose (HR 5/5) in conjunction with the FPLC system. All chromatographies were run in TEAD buffer. For elution of the enzyme from MonoQ a gradient of 0–500 mM NaCl and from Phenyl-Superose a gradient of 1.2–0 M (NH_4_)_2_SO_4_ were used, respectively. The purified enzyme was finally dialyzed against TEAD buffer.

#### 2.4.5. Purification of Proteasomes from Human Spleen, Cells, and Plasma

Purification of proteasomes from human erythrocytes and plasma was performed exactly as described by Zoeger et al. [32]. Briefly, “fraction II” was prepared from cell extract by use of DEAE-Sephacel, which was then used to obtain by ammonium sulphate (30–80% saturated with (NH_4_)_2_SO_4_) precipitation a proteasome-containing fraction. The enzyme was then purified by successive chromatography on DEAE-Toyopearl 650S, preparative Superose 6, and MonoQ. For all chromatographic TEAD buffer was used. Finally, the enzyme was subjected to affinity chromatography with an antibody to subunit *α*3 as ligand, as described elsewhere [32], and was then dialysed against TEAD buffer.

Normal human spleen tissue purchased from Enzo Life sciences Ltd.

#### 2.4.6. Two-Dimensional Polyacrylamidegel Electrophoresis (2D-PAGE)

Preparation and performing 2D-PAGE with purified proteasomes from BAL supernatant of ARDS patients in 8 × 10 cm gels were exactly done as described by Schmidt et al. [33]. Designation of proteasome subunits corresponded to that used by Schmidt et al. [33] and by Froment et al. [34] without applying the nomenclature of the minor subforms of the *α*- and *β*-subunits. Proteasome concentration of healthy subjects after purification was too low to allow additional 2-D PAGE electrophoresis.

#### 2.4.7. Proteasomal Activity

The proteasomal activity was measured fluorometrically in BAL supernatant in all ARDS patients and in all healthy controls using specific fluorogenic substrates and techniques previously described (19). We tested for peptidyl-glutamyl peptide-hydrolysing activity (PGPH) with 200 *μ*M benzoyloxycarbonyl-LLE-7-amido-4-methylcoumarin (Z-LLE-MCA), for trypsin-like activity (Try) with 200 *μ*M benzoyl-VGR-MCA (Bz-VGR-MCA), and for chymotrypsin-like activity (Chtr) with 100 *μ*M succinyl-LLVY-MCA (Suc-LLVY-MCA) as substrates (46, 47). All measurements were performed in duplicate and averaged for each subject. To describe the specific enzyme activity of extracellular proteasomes we used fluorogenic substrate cleavage (pmol/min × *μ*g).

#### 2.4.8. Analysis of Proteasome Subtypes

Purified 20S proteasomes from 5 pooled BAL supernatants of ARDS patients were separated by high-resolution anion exchange chromatography (in conjunction with a SMART-Chromatography System; Amersham Biosciences) on Mini Q equilibrated with TEAD-buffer exactly as described elsewhere [35]. Purification of 20S proteasome from pooled BAL of 5 healthy subjects turned out to be impossible due to the low 20S proteasome concentration in BAL supernatant.

#### 2.4.9. Lactate Dehydrogenase Activity in BAL Supernatant

Total (LDH_1_–LDH_5_) lactate dehydrogenase (LDH) activity was measured by a kinetic uv-test (Diaglobal GmbH, Berlin, FRG) using an optimized standard method (IFCC).

#### 2.4.10. Total Protein Concentrations in BAL Supernatant

Total protein concentration was determined after trichloroacetic acid (TCA) precipitation (5%), washing, and resolubilization according to Lowry using an autoanalyzer (Technicon) employing bovine serum albumin (BSA) as a standard.

### 2.5. Chemicals

All chemicals were of highest available or analytical grade. Water was deionized, distilled, and passed through a Milli-Q-System (Millipore, Witten) before use.

### 2.6. Statistical Analysis

Analyses were performed with SPSS, version 9 (SPSS, Inc., Chicago, USA). Continuous variables are presented as means ± standard deviation (SD). Nonparametric variables were compared by using the Mann-Whitney *U*-test, as indicated. Data are presented as median and range and were not normally distributed. Comparison of values of variables between groups (ARDS versus healthy subjects) was performed using the Mann-Whitney *U* test. Differences were regarded as statistically significant with an a priori alpha-error *P* of less than 0.05.

## 3. Results

**Figure 1:**
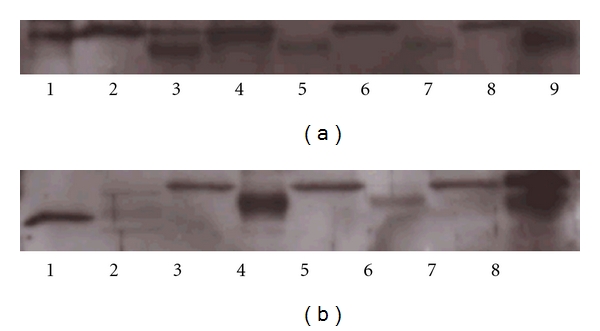
Western blots with a polyclonal antibody directed against LMP2 or LMP7 subunits of the immunoproteasome of samples of BAL supernatant and BAL cell pellet lysate obtained from ARDS patients. (a) LMP2 immunoproteasome protein was detected in both BAL supernatant and cell pellet in all ARDS patients. Lanes are identified as follows: Lane 1: 1 *μ*g immunoproteasome (human spleen); Lane 2: cell pellet ARDS patients 1; Lane 3: BAL supernatant ARDS patients 1; Lane 4: cell pellet ARDS patients 2; Lane 5: BAL supernatant ARDS patients 2; Lane 6: cell pellet ARDS patients 3; Lane 7: BAL supernatant ARDS patients 3; Lane 8: cell pellet ARDS patients 4; Lane 9: BAL supernatant ARDS patients 4. (b) LMP7 immunoproteasome protein was detected in both BAL supernatant and cell pellet in all ARDS patients. Lanes are identified as follows: Lane 1: 1 *μ*g immunoproteasome (human spleen); Lane 2: 1 *μ*g 20S standard proteasome (human erythrocyte); Lane 3: cell pellet ARDS patients 1; Lane 4: BAL supernatant ARDS patients 1; Lane 5: cell pellet ARDS patients 2; Lane 6: BAL supernatant ARDS patients 2; Lane 7: cell pellet ARDS patients 3; Lane 8: BAL supernatant ARDS patients 3.

**Figure 2:**
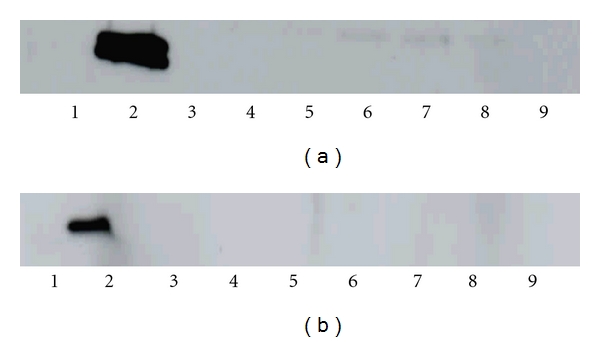
Representative western blots with a polyclonal antibody directed against LMP2 or LMP7 subunits of the immunoproteasome of samples of BAL supernatant obtained from healthy subjects. (a) LMP2 immunoproteasome protein could not be detected in BAL supernatant of any healthy subject. Lanes are identified as follows: Lane 1: 1 *μ*g 20S standard proteasome (human erythrocyte); Lane 2: 1 *μ*g immunoproteasome (human spleen); Lane 3: BAL supernatant healthy subject 1; Lane 4: BAL supernatant healthy subject 2; Lane 5: BAL supernatant healthy subject 3; Lane 6: BAL supernatant healthy subject 4; Lane 7: BAL supernatant healthy subject 5; Lane 8: BAL supernatant healthy subject 6; Lane 9: BAL supernatant healthy subject 7. (b) LMP7 immunoproteasome protein could not be detected in BAL supernatant of any healthy subject. Lanes are identified as follows. Lane 1: 1 *μ*g 20S standard proteasome (human erythrocyte); Lane 2: 1 *μ*g immunoproteasome (human spleen); Lane 3: BAL supernatant healthy subject 1; Lane 4: BAL supernatant healthy subject 2; Lane 5: BAL supernatant healthy subject 3; Lane 6: BAL supernatant healthy subject 4; Lane 7: BAL supernatant healthy subject 5; Lane 8: BAL supernatant healthy subject 6; Lane 9: BAL supernatant healthy subject 7.

Most important, all ARDS patients showed both LMP2 and LMP7 immunoproteasome proteins in the BAL supernatant and also in their cell pellet lysate (Figures 1(a) and 1(b)). In contrast, LMP7 and LMP2 were not detected in the BAL supernatant (Figures 2(a) and 2(b)) of any healthy subject. LMP2 was only detected in the cell pellet of healthy controls whereas LMP7 was not.

The molecular weight of the immunoproteasome positive protein bands in the western blots of the BAL cell pellet lysate from ARDS patients was greater than that in their BAL supernatants, suggesting that extracellular immunoproteasome protein-containing proteasomes are assembled from larger intracellular pro-proteins.

**Figure 3:**
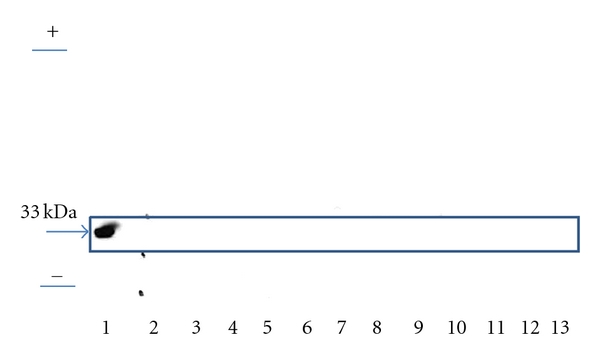
Representative Western blot with a polyclonal antibody directed against PA28 in BAL supernatant of twelve patients with ARDS. PA28 protein could not be detected in the BAL supernatant of ARDS patients. Start and front of the gel were marked as + and −. Lanes are identified as follows: Lane 1: 1 *μ*g PA28 (standard); Lane 2–13 BAL supernatant of twelve ARDS patients.

PA28 could neither be detected in BAL supernatants of all patients with ARDS nor in healthy controls.  Figure 3 shows a western blot with an antibody directed against the PA28 activator.

**Figure 4:**
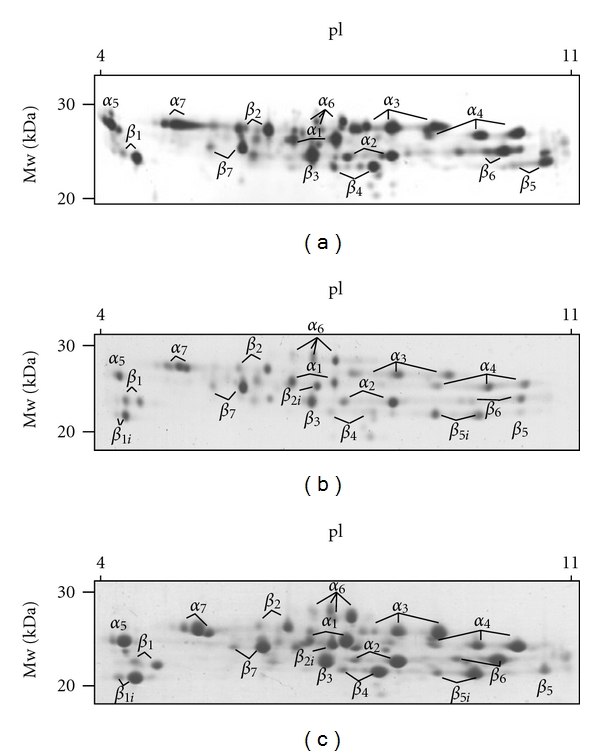
2D-PAGE of purified 20S proteasomes from (a) red cells (5 *μ*g), (b) BAL-supernatant (20 *μ*g) from ARDS patients, and (c) spleen (30 *μ*g). Detection of protein spots was performed by silver staining and Coomassie BB G250, respectively. Standard 20S proteasome was exclusively detected in red cells (a). Samples of human spleen and of the BAL supernatants from ARDS patients showed both standard and immunoproteasome proteins (panels (b) and (c)).

Purification and 2-D gelelectrophoresis of the BAL supernatant from ARDS patients showed 20S proteasomal core proteins (Figure 4(a)). Immunoproteasome subunits *β*_1i_ (LMP2), *β*_2i_ (MECL-1), and *β*_5i_ (LMP7) were detected in the two-dimensional polyacrylamide gelelectrophoresis (Figure 4) confirming the data derived from the western blots. Like BAL supernatant from ARDS patients samples of splenic tissue, but not human red cells, revealed immunoproteasome subunits.

Comparison of the specific activities of purified proteasome (Table 2) from pooled BAL supernatant of healthy controls and of ARDS patients showed a lower proteasomal activity in ARDS patients but also a different ratio of the individual proteasomal enzyme activities (Table 2) suggesting a change of proteasomal subunit composition. With a ratio of peptidyl-glutamyl peptide-hydrolysing activity (PGPH) to trypsin-like activity (Try) of 11.2 versus 14.6, a ratio of chymotrypsin-like activity (Chtr) to trypsin-like activity of 33 versus 14.5, and a ratio of the chymotrypsin-like activity to the peptidyl-glutamyl peptide-hydrolysing activity (Chtr/PGPH: 2.95 versus 0.99) these activity ratios were different in ARDS patients when compared to healthy controls.

**Figure 5:**
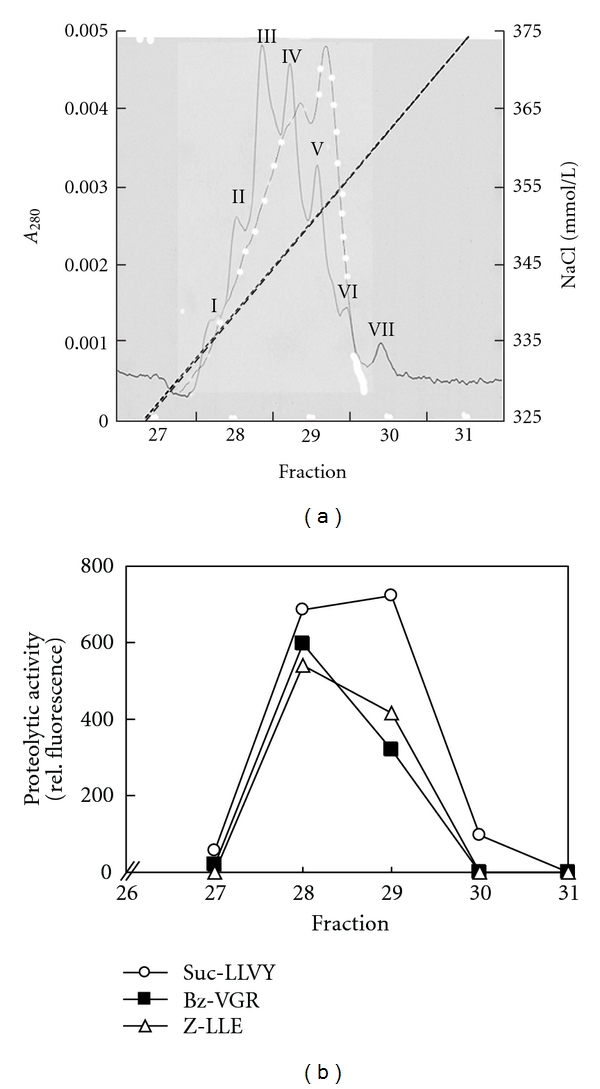
Subtype pattern of the extracellular alveolar proteasome of patients with ARDS (continuous line) and of plasma from healthy subjects (white points). (a) 20 *μ*g of 20S proteasome from pooled BAL supernatant of five ARDS patients were purified, subjected to chromatography on Mini Q, and separated into their subtypes by elution with increasing concentrations of NaCl. Subtypes detected by absorption at 280 nm are designated by roman figures (I–VII) according to the order of their elution from the column. All subtypes elute at NaCl concentrations (dashed line) between 330 and 370 mmolNaCl/L, and only this detail of the chromatograms is shown. (b) All collected fractions of the subtype pattern chromatography of the extracellular alveolar proteasome of patients with ARDS were measured by the highly specific proteasomal fluorogenic peptides Suc-LLVY-AMC (open points), BZ-VGR-AMC (black rectangle), and Suc-LLE-AMC (Z-LLE-AMC) (open triangle). Only in the fractions 28–30, proteasomal enzyme activity could be observed. Analysis of alveolar proteasome revealed a new proteasomal subtype pattern in the extracellular alveolar space of ARDS patients that differs from that of healthy subjects' plasma, suggesting that the extracellular alveolar proteasome in ARDS does not derive from plasma.

Chromatography (Figure 5) of a pooled sample of BAL supernatants from 5 ARDS patients revealed a new proteasomal subtype pattern with distinct numbers and proportions of seven peaks (I–VII) unlike that of human circulating plasma proteasome. In fact, since the alveolar subtype pattern seen in ARDS patients was not even similar to the subtype patterns found in erythrocytes, platelets, monocytes, and T lymphocytes (32), respectively, the extracellular alveolar proteasome found in ARDS patients is unlikely to derive from the blood stream.

In contrast to the BAL supernatant of healthy individuals, the plasma and the BAL cell pellet of all healthy subjects and of all ARDS patients showed both LMP2 and LMP7 proteins in the western blots (data not shown).

Total proteasome concentration in BAL supernatants of ARDS patients was higher (971 ± 1116 ng/mL) compared to healthy subjects (59 ± 25; *P* < 0.001) (Table 3), and all fluorogenic substrates were hydrolyzed by BAL supernatants of ARDS patients (Suc-LLVY-AMC: 3.1 ± 6.2 pkat/mg; Bz-VGR-AMC: 1.8 ± 2.5; Z-LLE-AMC: 0.8 ± 1.1) and of healthy subjects (Suc-LLVY-AMC: 7.3 ± 3.7 pkat/mg; Bz-VGR-AMC: 5.6 ± 3.2; Z-LLE-AMC: 2 ± 1.2), with inhibition by epoxomicin (*P* = 0.0001).

There was no significant correlation (*P* = 0.16) in ARDS patients between proteasome concentration in BAL supernatant and in their plasma. In addition, there was no correlation between LDH activity and proteasome concentration in BAL supernatant (*P* = 0.21), or between BAL cell count and proteasome concentration in BAL supernatant (*P* = 0.26), ruling out cell lysis as a major source of proteasome in the extracellular alveolar space.

Our patients by any criteria had severe ARDS (Table 1) and also showed marked physiological derangements, as indicated by a high simplified acute physiology score and sepsis-related organ failure assessment.

## 4. Discussion

Our data show that the extracellular alveolar space in ARDS patients contains (1) an altered proteasomal composition with a distinct proteasomal subtype pattern, and (2) immunoproteasome proteins, that is, a different type of proteasome when compared to healthy subjects. Most likely, these alterations are evoked by pulmonary inflammation.

Intracellularly, three subpopulations of 20S proteasomes are known, that is, standard proteasome, immunoproteasome, and intermediate-type proteasomes. These subpopulations can only be separated and characterized by high-resolution anion exchange chromatography, as used in our study, and not by ELISA or western blotting techniques.

Detection of the immunoproteasomal subunits LMP 2 and LMP 7 in the extracellular alveolar space in all ARDS patients but not in healthy controls was associated with a change in the ratio of proteasomal enzyme activities as revealed following purification. This is consistent with reports that intermediate-type proteasomes or immunoproteasomes, at least in cell cultures, show an altered ratio of peptidyl-glutamyl peptide-hydrolyzing to trypsin-like activity and of chymotrypsin-like to trypsin-like activity when compared to the standard 20S proteasome [11, 13, 14, 36, 37]. Presumably, this is caused by a decrease in size and charge of the S1 pocket of *β*_i1_ as compared to that of *β*_1_ [38]. Thus, the observed change of proteasomal composition and activity in BAL supernatant of ARDS patients may be caused by replacement of standard 20S proteasome proteins by catalytic subunits *β*_1i_ (LMP2), *β*_2i_ (MECL-1), and *β*_5i_ (LMP7) that are incorporated into a newly synthesized intermediate type and/or immunoproteasome.

Despite detection of immunoproteasome proteins LMP 2 and LMP 7 by western blot and of MECL-1 by 2-D gelelectrophoresis it remains unclear whether it is pure immunoproteasome and/or intermediate-type proteasomes that are found in the extracellular alveolar space of ARDS patients. However, data obtained in cells [32] suggest that the proteasomal subtype pattern seen in BAL supernatant of our ARDS patients represents intermediate-type proteasome as a dominant proteasome fraction. The higher molecular weight of the Immuno *β* catalytic subunits in the cell pellet lysate of ARDS patients presumed the existence of proproteins as described elsewhere [39–41]. These findings suggest that the Immuno *β* catalytic subunits were built in the cell pellet, and the completed immunoproteasomes were transported into the alveolar space. This mechanism of extracellular transport of the immunoproteasome is unclear and further work had to be done to clarify this question.

In any case, that immunoproteasome proteins were detected in the BAL supernatant of ARDS patients but not in healthy individuals, which likely represents a biological reaction in response to alveolar inflammation. ARDS results in a marked proinflammatory response with high IFN-*γ* and TNF-*α* [21, 42, 43] concentration in the alveolar space. While we did not measure alveolar cytokine concentrations one may speculate that high IFN-*γ* concentrations induce the assembly of immunoproteasome proteins. In this context, the greater molecular weight of the immuno *β* catalytic subunits found in the cell pellet lysate of ARDS patients suggests the existence of immunoproteasome pro-proteins (13–15) that by a yet undefined mechanism apparently gain access to the extracellular space.

In this study, we identified for the first time a new proteasomal subtype pattern in the alveolar space of ARDS patients that differs from that of proteasomes in blood cells. Therefore, the extracellular alveolar immunoproteasome and/or intermediate-type proteasome found in ARDS patients is unlikely to derive from cytolysis of blood cells and sequestration of their contents into alveoli across leaky endothelial and epithelial barriers. This is supported by the finding that no significant correlation between the proteasome concentration in plasma and in BAL supernatant was seen. Thus, while endothelial and epithelial damage as well as basement membrane destruction is a feature of ARDS [20, 44] extravasation of circulating proteasomes alone cannot be responsible for the presence of extracellular alveolar 20S proteasomes.

By the same token, it is unlikely that alteration of proteasomal composition in the alveolar space in ARDS patients resulted from lysis of cells of the alveolar wall. This appears to be ruled out by the fact that PA28 proteasomal caps, normally present intracellularly, were not found in western blots from BAL supernatant of patients with ARDS. In addition, masked PA28 proteasomal caps (by proteins or protein complexes) might not be accessible using western blot analysis so that this conclusion has to be verified by MS analysis. Furthermore, no significant correlation between total proteasomal concentration in BAL supernatant and LDH activity, a marker of cell lysis, or with the BAL cell count was observed. Thus, the presence of immunoproteasome proteins likely relates to the inflammatory process in lung tissue rather than to cell lysis.

Since no 19S and PA28 proteasomal cap proteins were detected by western blot of BAL supernatant, 26S proteasome and/or hybrid proteasome were not present in the alveolar space of patients with ARDS. However, since the detection limit of our method is in the range of 0.5–1 *μ*g protein/*μ*L we cannot exclude the presence of lesser extracellular concentrations of 26S proteasome.

Our data showing the presence of immunoproteasome proteins and a distinct proteasomal subtype pattern in BAL supernatant from patients with ARDS extend our previous work [19] reporting increased total proteasome concentrations but lesser proteasomal activities when compared to healthy subjects.

Different types of proteasomes are known to have different cleavage repertoires [45] and to yield different peptides for antigen presentation [16]. Possibly, a function of the extracellular immunoproteasome, evoked by inflammation, could be to cleave epitopes different from that of the standard 20S proteasome. It is unknown which extracellular proteins are degraded by the standard proteasome and which ones by the immunoproteasome or the intermediate-type proteasome. However, the presence of immunoproteasome proteins may suggest an altered extracellular protein degradation [26]. In any case, the presence of immunoproteasome proteins in the BAL supernatant of ARDS patients raises the provocative question whether antigen processing and hence part of the immunological response could also take place in the extracellular alveolar space.

To our knowledge, this study is the first to address the presence of immunoproteasome proteins in lung disease and the activity of extracellular alveolar proteasome in ARDS patients. Fluorogenic substrates, used in combination with epoxomicin, the most potent, selective, and irreversible proteasome inhibitor currently available, and an ELISA are accepted methods for analyzing proteasomal existence and activity [30, 46, 47]. In this study, we used an ELISA technique for the measurement of proteasomal concentration in the BAL supernatant. This technique does not allow to discriminate quantitatively between the 20S proteasome and the immunoproteasome. The western blots directed against LMP2 and LMP7, however, showed high signal intensity of the immunoproteasome proteins, likely reflecting a high concentration of immunoproteasome proteins in the BAL supernatant, in patients with ARDS but not in healthy controls.

It is conceivable, therefore, that quantitative immunoproteasome measurements in BAL might provide discrimination between disease activity, clinical scores, predictable survival, and efficacy of therapy. Obviously, this should be addressed in further studies.

In summary, we identified immunoproteasome proteins in the extracellular alveolar space of patients with ARDS, which are absent in healthy controls, and we discovered a distinct, previously undescribed alveolar proteasome subtype pattern that differs from the 20S proteasomes found in various blood cells. This may alter cleavage of alveolar proteins existing in the alveolar space during pulmonary inflammation seen in ARDS.

## Figures and Tables

**Table 1:** Clinical characteristics of ARDS patients.

PaO_2_/FiO_2_ ratio [mmHg]	82 ± 30
Positive end-expiratory pressure (PEEP) [mbar]	16 ± 4
Venous admixture [%]	45 ± 11
Compliance [mL/mbar]	26 ± 15
Lung injury score (LIS)	3.4 ± 0.4
ECMO therapy [%]	50
In-hospital mortality [%]	53.6
Simplified acute physiology score (SAPS)	63.5 ± 13.6
Sepsis-related organ failure assessment (SOFA)	15.1 ± 3.2

Means ± SD from 28 patients with ARDS. Data were obtained within 24 hours of admission.

**Table 2:** Specific activities of proteasomes isolated from healthy controls and ARDS patients.

	Chtr (pmol/min *μ*g)	Try (pmol/min *μ*g)	PGPH (pmol/min *μ*g)	Chtr/PGPH	PGPH/Try	Chtr/Try
Healthy controls	24.31	0.73	8.22	2.95	11.2	33.3
ARDS patients	9.87	0.68	9.93	0.99	14.6	14.5

Proteolytic activities of purified 20S proteasome from BAL supernatant of healthy controls and of ARDS patients, as measured with specific proteasomal fluorogenic substrates. BAL supernatants were pooled from 5 healthy subjects and from 5 ARDS patients, respectively. The ratio of enzyme activities differs between ARDS patients and healthy subjects, suggesting a rearrangement of proteasomal subunit composition.

PGPH: peptidyl-glutamyl peptide-hydrolysing activity; Try: trypsin-like activity; Chtr: chymotrypsin-like activity.

**Table 3:** Characteristics of BAL in ARDS and healthy subjects.

	ARDS patients (*n* = 28)	Healthy subjects (*n* = 10)	*P* value
Proteasome concentration in BAL supernatant [ng/mL]	971 ± 1116	59 ± 25	<0.001
Proteasome concentration in plasma [ng/mL]	2855 ± 2422	348 ± 126	<0.001
Suc-LLVY-AMC proteasome activity in BAL supernatant [pkat/mg]	3.1 ± 6.2	7.3 ± 3.7	<0.001
BZ-VGR-AMC proteasome activity in BAL supernatant [pkat/mg]	1.8 ± 2.5	5.7 ± 3.2	<0.001
Suc-LLE-AMC proteasome activity in BAL supernatant [pkat/mg]	0.8 ± 1.1	2 ± 1.2	0.002
Total protein concentration in BAL supernatant [mg/mL]	3.8 ± 6.4	0.06 ± 0.01	<0.001
Albumin concentration in BAL supernatant [mg/mL]	1.5 ± 3	0.03 ± 0.01	0.0011
LDH in BAL supernatant [U/L]	342 ± 779	28 ± 9.7	0.024
LDH in plasma [U/L]	821 ± 1104	184 ± 53	<0.001
Cell count in cell pellet [10^6^/mL]	330 ± 994	8.6 ± 2.5	0.007
Macrophages [%]	29.1 ± 27.4	92.6 ± 3.4	<0.001
Neutrophile granulocytes [%]	65.1 ± 27.7	2.8 ± 2.5	<0.001
Lymphocytes [%]	5.1 ± 7.9	6.3 ± 2.9	0.056

Data are means ± SD.
